# The pancreatic juice length in the stent tube as the predicting factor of clinical relevant postoperative pancreatic fistula after pancreaticoduodenectomy

**DOI:** 10.1097/MD.0000000000008451

**Published:** 2017-11-03

**Authors:** Hangyan Wang, Dianrong Xiu, Ming Tao

**Affiliations:** Department of General Surgery, Peking University Third Hospital, Haidian District, Beijing, P.R. China.

**Keywords:** pancreatic fistula, pancreatic juice, pancreaticoduodenectomy

## Abstract

Several risk factors for pancreatic fistula had been widely reported, but there was no research focusing on the exocrine output of remnant gland.

During the study period of January 2015 to September 2016, 82 patients accepted pancreaticoduodenectomy (PD, end-to-end dunking pancreaticojejunostomy with internal stent tube). All the data were collected, including preoperative medical status, operative course, final pathology, gland texture, pancreatic duct diameter, size of the stent, length of pancreatic juice in the stent tube, width of the pancreatic stump, diameter of the jejunum and the status of postoperative pancreatic fistula (POPF). POPF was defined according to International Study Group of Pancreatic Fistula criteria.

The diameter of pancreatic duct in the POPF group was significantly smaller than that in the group without POPF (1.99 vs 2.90 mm, *P* = .000). The length of pancreatic juice in the stent tube in the POPF group was significantly longer than that in the group without POPF (18.04 vs 6.92 cm, *P* = .014). There were more pancreatic ductal adenocarcinoma cases and hard glands in the group without POPF. The length of pancreatic juice in the clinically relevant postoperative pancreatic fistula (CR-POPF) group was significantly longer than that in the grade A group (32.4 vs 9.21 cm, *P* = .000). Multivariate analysis identified gland texture and length of pancreatic juice as independent predictors for pancreatic fistula. Multivariate analysis also identified the length of pancreatic juice as an independent predictor for CR-POPF.

The length of pancreatic juice in the stent tube might be a useful predictive factor of POPF after PD, especially for CR-POPF.

## 1 Introduction

The development in surgical technique and postoperative management technology have reduced the rates of mortality in the patients undergoing pancreaticoduodenectomy (PD), while the rate of clinically relevant postoperative pancreatic fistula (CR-POPF) continued to persist at approximately 15%.^[1–4]^ The CR-POPF is one of the most important life-threatening complications that could lead to intraabdominal abscess, hemorrhage, and sepsis.^[5,6]^ Therefore, perioperative assessment of patients at high risk of CR-POPF is very important, which may help the surgeons to adjust postoperative management.

Previous reports have evaluated the perioperative factors that influence the incidence of postoperative pancreatic fistula (POPF), such as pathology, gland texture, pancreatic duct, body mass index (BMI), and amylase level in the drainage fluid.^[4,7–12]^ Up to now, hard gland texture and dilated pancreatic duct have been widely accepted as the protective factors of POPF. However, there were some obvious limitations to the previous factors such as gland texture.

The exocrine output from the pancreas was widely implicated as the initial cause of fistula.^[13–15]^ The exocrine function of remnant gland played an important role in the development of POPF. However, there was no such research focusing on the exocrine output of remnant gland, which might influence the incidence of POPF. So, the aim of this research was to study the exocrine output of remnant gland and tried to reveal the relationship between the exocrine output and POPF.

## 2 Methods

The study was a retrospective analysis of 82 patients who underwent PD from January 2015 to September 2016 in the General Surgery Department of Peking University Third Hospital by the same staffs. This study was approved by the Peking University Third Hospital Medical Science Research Ethic Committee, and all patients signed informed consents. The reconstruction after the Whipple procedure was done by the Child method with end-to-end dunking pancreaticojejunostom. Silicone tubes were inserted as stent tubes in all cases regardless of the texture of the pancreas or the diameter of the pancreatic duct. The pancreatic juice was excreted into the stent tube. Six minutes after the insertion, the length of pancreatic juice in the tube was measured and then a 20 cm tube stent was left in the anastomosis as the internal pancreatic duct stent.

Drainages were placed in all patients during the surgeries. Prophylactic somatostatin analogue (octreotide acetate, 0.1 mg, 3 times daily, Sandostatin, Novartis, Switzerland) was used in all cases for 3 days after the operation.

All data were collected, including preoperative medical status, operative course, final pathology, gland texture, pancreatic duct diameter, size of the stent, length of pancreatic juice in the stent tube, width of the pancreatic stump, and diameter of the jejunum. We defined POPF according to International Study Group of Pancreatic Fistula (ISGPF) definition as the output from an operative drain on or after postoperative day (POD) 3 with a drain amylase content higher than 3 times the serum, graded as A, B, or C.^[16]^ Grade A was considered to be transient POPF without clinical impact. Grade B and C required changes in management or adjustment in the clinical pathway and were defined as CR-POPF.

Values were presented as means ± standard deviation. χ^2^ tests were used to examine categorical independent variables. A Student *t* test was used to compare variables with a normal distribution; variables not normally distributed were analyzed using the nonparametric Wilcoxon–Mann–Whitney test. Multivariate analyses were performed using logistic-regression analysis. A *P* value less than .05 was considered statistically significant. All statistical tests were performed using the SPSS 19.0 statistical software.

## 3 Results

During the study period of January 2015 to September 2016, 82 patients were scheduled. Characteristics of the patients, intraoperative data, and postoperative course are summarized in Table 1. Of these 82 patients, 42 (51.2%) patients were diagnosed as POPF according to the ISGPF criteria. In all the POPF cases, 26 cases were grade A, 14 cases were grade B, and 2 cases were grade C.

**Table 1:**
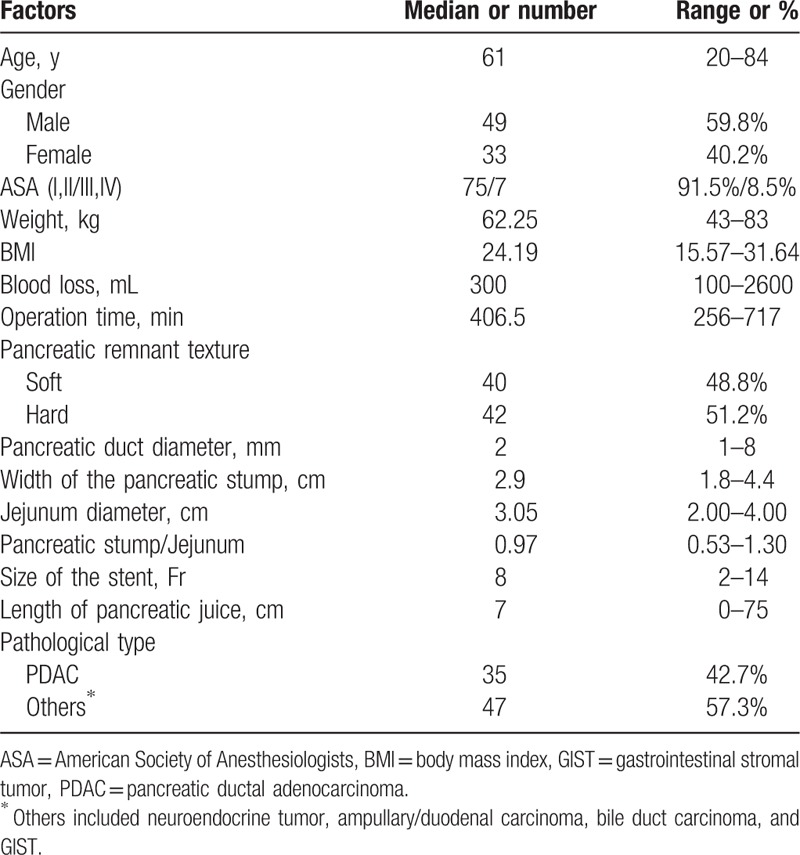
Patient characteristics.

Preoperative demographic characteristics and surgical characteristics associated with POPF are shown in the Table 2. There were 40 cases in the POPF group and 42 cases in the non-POPF group. The pancreatic duct diameter in the POPF group was significantly smaller than that in the non-POPF group (1.99 vs 2.90 mm, *P* = .000). The size of the stent in the POPF group was significantly smaller than that in the non-POPF group (6.67 vs 8.95 Fr, *P* = .000). The pancreatic stump in the POPF group was significantly wider than that in the non-POPF group (3.02 vs 2.77 cm, *P* = .000). The jejunum diameter in the POPF group was significantly larger than that in the non-POPF group (3.23 vs 2.88 cm, *P* = .000). The length of pancreatic juice in the stent tube in the POPF group was significantly longer than that in the non-POPF group (18.04 vs 6.92 cm, *P* = .014). There were more pancreatic ductal adenocarcinoma cases and hard glands in the non-POPF group. There were no significant differences in gender, age, weight, BMI, American Society of Anesthesiologists status, blood loss, operation time, and pancreatic stump/Jejunum between the 2 groups. Multivariate analysis identified gland texture and length of pancreatic juice as independent predictors for POPF (Table 3). Multivariate analysis also identified the length of pancreatic juice as an independent predictor for CR-POPF (odds ratio, 1.128; 95% confidential interval, 1.041–1.223; *P* = .003).

**Table 2:**
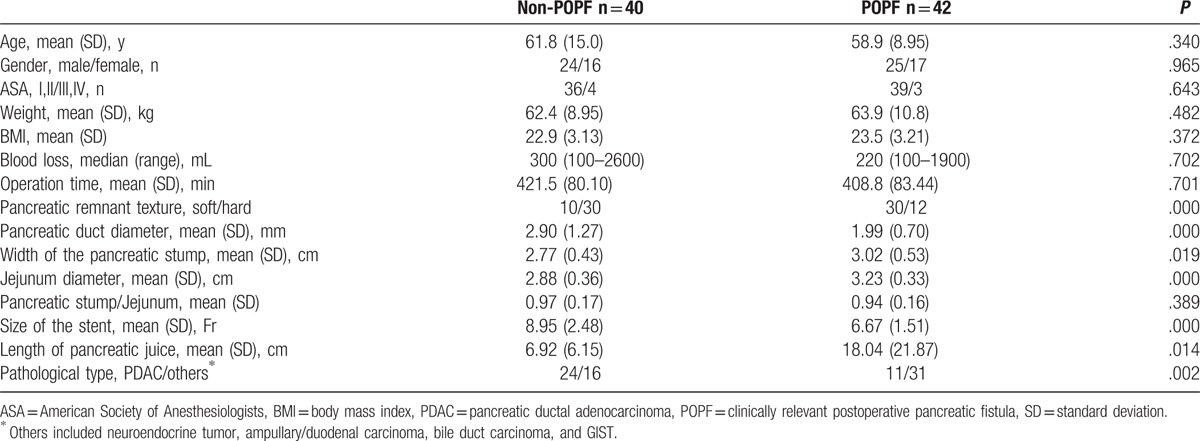
Comparisons between POPF group and non-POPF group.

**Table 3:**
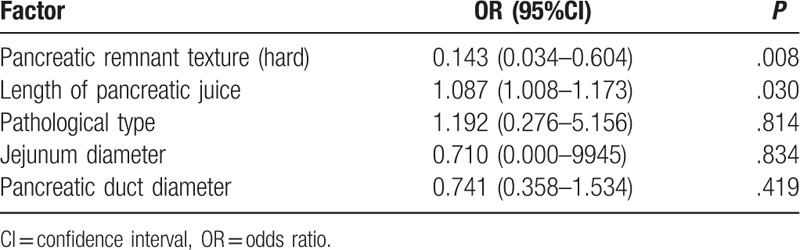
Predictive factors of postoperative pancreatic fistula by multivariate analysis.

Patients’ demographic characteristics and surgical data associated with POPF are shown in the Table 4. There were 26 patients in the grade A group and 16 patients in the CR-POPF group. The weight in the CR-POPF group was significantly higher than that in the grade A group (69.8 vs 60.8 kg, *P* = .016). The length of pancreatic juice in the CR-POPF group was significantly longer than that in the grade A group (32.4 vs 9.21 cm, *P* = .000). There were no significant differences in gender, age, BMI, American Society of Anesthesiologists status, blood loss, operation time, pancreatic stump width, jejunum diameter, pancreatic stump/Jejunum, pancreatic duct diameter, size of the stent, pathological type, and gland texture between the 2 groups.

**Table 4:**
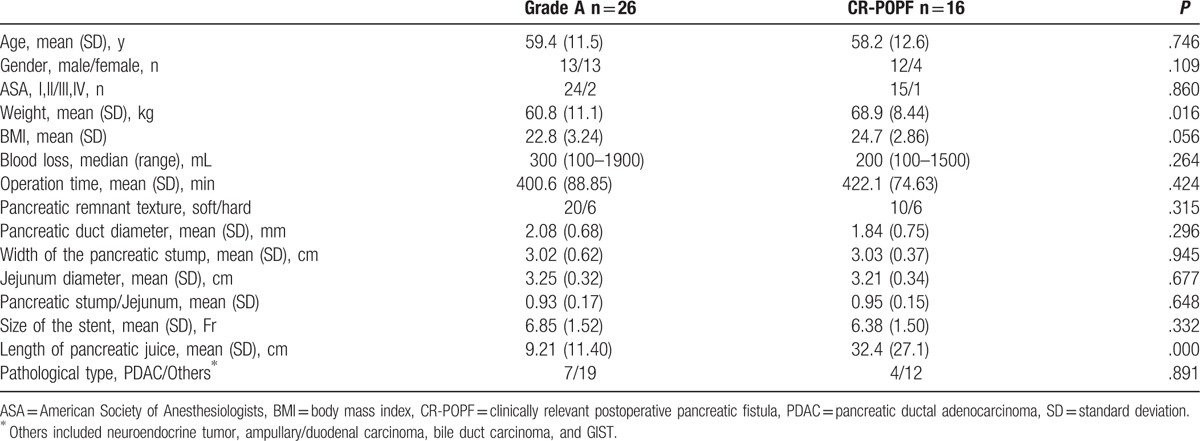
Comparisons between grade A group and clinically relevant postoperative pancreatic fistula (grade B and C) group.

In the POPF group, the length of pancreatic juice was the independent predictor for CR-POPF (odds ratio, 1.096; 95% confidential interval, 1.020–1.178; *P* = .013; Table 5).

**Table 5:**
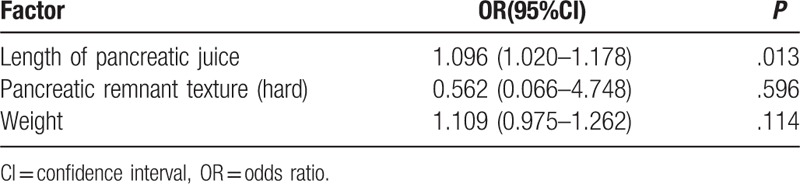
Predictive factors of clinically relevant postoperative pancreatic fistula by multivariate analysis in the postoperative pancreatic fistula group.

## 4 Discussion

Since the ISGPF classification was declared in 2005, it has been widely accepted. However, the ISGPF classification was a reporting system not a predicting system. So, after the diagnosis of POPF, we still want to know whether the POPF will develop toward a complicated fistula that needs specific intervention or whether it will heal spontaneously without further intervention. How can one identify the patient with a pancreatic fistula that will probably develop complications, as opposed to the patient who can be safely discharged with a drain and treated on an outpatient basis? How to distinguish the “high risk” and “low risk” patients as early as possible and to make decision of taking critical treatment for high risk patients and avoiding over medicalization for low risk patients? The essential question for the management of POPF still is whether one can, in the early period after pancreatic surgery, distinguish CR-POPF (grade B and C), which need more intervention, from transient pancreatic fistula (grade A).^[17]^ The assessment of the risk of CR-POPF is important for the surgeons to make a different decision of postoperative management such as the timing of drainage removal and diet recovery. The risk factors for POPF and CR-POPF have been reported but there were still controversies.^[18,19]^

Pancreatic texture and pancreatic duct diameter were the most widely recognized risk factors for POPF. Soft pancreas was associated with higher POPF rate and led to a 10-fold increased risk of POPF versus hard gland.^[10]^ The size of pancreatic duct also has been implicated as a major predictor of POPF, particularly when the diameter of main pancreatic duct was less than 3 mm.^[20,21]^ Another widely used predicting tool was the fistula risk score, including 4 endogenous and operative risk factors identified at the point of anastomotic reconstruction. These factors include gland texture, pathology, pancreatic duct diameter, and intraoperative blood loss.^[22]^ Fistula risk score was considered to be a strong and comprehensive predictive system for CR-POPF development.^[22–24]^

As we can see, the gland texture was the widely used predicting factor for POPF or CR-POPF, but the limitation of gland texture was obvious. The gland texture was a subjective factor which depended on the surgeon's experience. There was no unitary standard for gland texture especially in the moderate texture glands and how can one surgeon to distinguish the gland texture if it was neither too hard nor too soft. Previous predictive factors such as gland texture and duct width mainly focused on the structure or anastomosis difficulty. We still lacked factors to assess the exocrine function of remnant pancreas, which might be more important in the development of CR-POPF. One good example for this point was the patients with chronic pancreatitis. The POPF was less common in the patients who accepted PD because of chronic pancreatitis. Chronic pancreatitis often meant hard gland texture and dilated pancreatic duct which facilitated the anastomosis, but the exocrine deficiency caused by the chronic pancreatitis might be another reason for the protective influence of chronic pancreatitis.

In our study, we tried to analyze the factors which influenced the development of POPF. As previous researches, we also found the pancreatic duct diameter and size of the stent were significantly smaller in the POPF group and there were more pancreatic ductal adenocarcinoma cases and hard glands in the non-POPF group. Multivariate analysis also identified gland texture as independent predictors for POPF. However, there were no differences of these factors between the CR-POPF group and grade A group, and multivariate analysis identified the length of pancreatic juice as the only independent predictor for CR-POPF. In our study, the pancreas texture was identified as the predicting factor of POPF but not for CR-POPF. The length of pancreas juice in the stent tube was the predicting factor of POPF and CR-POPF. For this consideration the length of juice might be a better factor than previous factors such as gland texture and pancreatic duct diameter.

The soft gland and nondilated pancreatic duct usually caused difficult and unsatisfied anastomosis, which might mean more POPF risks. That was why these factors were considered as the risk factors of the POPF. But the development of the CR-POPF was a complex pathophysiology procedure. The leakage of pancreatic juice was the initial factors, but the exocrine function and inflammation factors played another important role in the development of the CR-POPF. The exocrine function factors included the quality of the pancreatic juice, the enzymes in the pancreatic juice; the activated status and the aggressive ability of the enzymes. The exocrine function was obviously ignored previously and we needed direct factors to assess the exocrine function. The length of pancreatic juice could partially reflect the exocrine function of remnant pancreas and may be a useful predictor for CR-POPF.

The exocrine output of remnant pancreas was widely implicated as the initial cause of fistula. The underlying process is the continuous leakage of caustic proteases and lipolytic enzymes, with significant local consequences (abscess, pancreatic fistula, acute pancreatitis, and pseudoaneurysm) and systemic sequelae (sepsis, shock, and pulmonary insufficiency).^[25]^ So, it was reasonable and important to assess the exocrine output of remnant pancreas to predict the severity of POPF. The length of pancreatic juice in the stent was a direct index for the exocrine function of remnant pancreas. It also might be a comprehensive and objective predicting factor for CR-POPF. In the same situation of unsatisfactory anastomosis, the higher exocrine output of remnant pancreas meant more leakage and higher risk of CR-POPF. Tumors in the head of the pancreas usually caused obstructive pancreatitis, which meant dilated pancreatic duct and hard gland. The dilated pancreatic duct and hard and fibrous gland were also considered to be the phenomena of the deficiency of pancreatic exocrine function. The dilated pancreatic duct and hard gland were also considered to be protective factors against POPF. These factors could be summarized in a single objective and reproducible parameter through the length of the pancreatic juice in the stent tube, which might be a better parameter than these subjective or poor-standardized factors such as gland texture. Better exocrine function of pancreas (soft gland) and thinner pancreatic duct caused longer pancreatic juice in the stent tube, which meant more risk of CR-POPF. This might be the reason why the length of the pancreatic juice had the predictive capacity for CR-POPF. In our research, we also confirmed the length of pancreatic juice as the only independent predictor for CR-POPF. Of course this point needed more detailed researches.

The limitations of the current study are its retrospective nature and the small number of patients, and all the results were gained from our reconstruction method, dunking pancreatojejunostomy with internal stenting. Nevertheless, all the patients were operated by the same team at the same institution with the same strategies, which made it homogeneous and added strength to our results. Besides, in our study we just focused on the quantity of exocrine output of remnant pancreas (the length of the pancreatic juice), the quality of the exocrine (the enzymes or proenzymes in the juice) might be another important question.

In conclusion, the length of pancreatic juice could predict postoperative POPF after PD, especially CR-POPF.
